# Communication or Toxicity: What Is the Effect of Cycloheximide on Leaf-Cutting Ant Workers?

**DOI:** 10.3390/insects8040126

**Published:** 2017-11-21

**Authors:** Kátia Kaelly Andrade Sousa, Roberto da Silva Camargo, Luiz Carlos Forti

**Affiliations:** Laboratório de Insetos Sociais-Praga, Departamento de Proteção Vegetal, Faculdade de Ciências Agronômicas/UNESP, Caixa Postal 237, Botucatu, SP 18603-970, Brazil; katiakaelly@gmail.com (K.K.A.S.); luizforti@fca.unesp.br (L.C.F.)

**Keywords:** communication, leaf-cutter ant, fungicide

## Abstract

Leaf-cutting ants are insects that use plant material to grow fungus from which they feed. These fungus-growing ants perform various behavioral activities to establish an environment conducive to the fungus. Among these behaviors are activities that can serve to detect materials harmful to the colony, such as licking, scraping, chopping, etc. However, there are substances that may not be detected as harmful to the fungus on first contact. Cycloheximide (CHX) is one such substance, described as a fungicide that inhibits the synthesis of proteins in eukaryotes, although its mechanism of action remains unclear. The present study aimed at evaluating the behavioral changes of worker ants, time carrying orange pellets, waste deposition and mortality, when subjected to seven days of CHX-incorporated pellets and another seven days of CHX-free pellets. The fungicide adversely and structurally affected the leaf-cutter ant colonies. Their behavior went through changes, such as an increase in pellet-licking frequencies and cleaning among the ants from the third day onward. Moreover, there was an increase in time carrying orange pellets, as well as in the mortality of workers during CHX incorporation.

## 1. Introduction

Leaf-cutting ants are neotropical social insects that present a symbiotic relationship with fungus, in which the ants provide favorable conditions for the growth of the symbiotic fungus, a source of food for the colony, this being a beneficial relationship [1,2]. Leaf-cutter ants use several strategies to provide the fungus garden with healthy growth. Once the plant material has been selected and transported to the fungus chambers, this material goes through several processing stages, ranging from cutting, to its incorporation into the fungus garden [3].

This compulsory relationship prompted workers to learn to reject material containing substances harmful to the symbiotic fungus. This was experimentally observed with the supply of pellets with cycloheximide (CHX), an antibiotic produced by *Streptomyces griseus*, which inhibits protein synthesis. CHX is also known as a growth inhibitor of many yeasts and fungi in colonies of *Atta laevigata* Smith, presenting an approximately 48-h acceptance of pellets and then prompting their rejection, even in the absence of fungicide [4]. According to the authors, the workers communicate among themselves not to accept the forage material, this being a response produced by the “stressed” fungus, which produces a semi-chemical that prevents any incorporation of the forage material into the fungus garden. On the other hand, in a study with *A. sexdens rubropilosa* Forel, this communication was not observed, with only a possible communication between workers [5]. With the *Acromyrmex lundi* Guering species, the supply of CHX, together with the leaves, led to a similar result, in which the treated material was rejected 10 h after its incorporation and was maintained for nine weeks [6].

Subsequently, CHX was used in studies with leaf-cutting ants that involved a long-term olfactory memory process. These studies covered plant selection by gardener ants, in which waste particles removed from the garden to the garbage could contain clues of inadequate plants or damaged fungus [7,8,9,10,11]. Fungicide infiltration into the leaf was experimentally induced and then offered to a colony of *Atta colombica* (Guerin) for the study of long-term olfactory memory; it was observed that the ants began rejecting the material for up to 18 weeks, indicating strong long-term rejection learning by forage workers [10]. It was also verified that the workers of the species *Acromyrmex ambiguus* Emery presented, after two days of learning, an increase in their mass of synaptic complexes. However, there was a reduction of mass to its initial state from the fourth to the fifteenth day, that is there was structural remodeling of the brain. This reorganization was triggered by the avoidance of long-term memory formation. This species also rejected material with CHX, which was latter incorporated into the fungus [11,12].

CHX is widely used for insects such as flies, crickets, butterflies and *Coleoptera*, among others, in studies involving the formation of long-term memory, toxicity of resistant genotypes, altered morphology, altered protein levels and neuronal destruction [13,14,15,16,17,18,19,20]. Within this broad field of study, substance activity tends to present a great variation of results, which manifest in different ways, depending on the studied order. However, its toxicity to leaf-cutting ant workers is not shown in any of these studies [4,5,6,7,8,9,10,11,12].

Given the above, on the intrinsic relationship between the leaf cutting ants and their symbiont fungus, a question arises: What is the effect of cycloheximide on leaf-cutting ant workers? Thus, this study proposed to identify the behavioral changes of workers during fungus garden cultivation with baits containing the synthetic fungicide CHX. In addition, we studied the worker mortality, garbage production, as well as the carrying of orange pellets and return rate of pellets.

## 2. Material and Methods

### 2.1. Studied Colonies

We used colonies of *Atta sexdens rubropilosa* to perform the experimental series, which were kept in the Social Insects Laboratory, UNESP, Botucatu. The twelve queen-right colonies, collected in March 2016, were one year old at the start of these experiments. Each colony had a container (length: 15 cm, width: 15 cm and height: 15 cm) with a fungus garden, and these are fed on *Acalypha* spp. and *Ligustrum* spp. (leaves and stems). The colonies were kept at a temperature of 24 ± 2 °C, relative humidity of 80% and a photoperiod of 12 h light.

#### 2.1.1. Preparation of Pellets

Pellets consisted of small granules made of orange peels, which were dehydrated at 50 °C for 72 h. The orange peels were from organic crops. We dehydrated and ground these peels into a powder, in a milling process. Subsequently, 1.8 g of this powder were homogeneously mixed with 0.2 g of carboxy methyl cellulose to which 0.025 g of CHX (Sigma-Aldrich, St. Louis, MO, USA) dissolved in 2.5 mL of water were added to produce the orange granules with cycloheximide. The matrix was placed in a 20-mL syringe to produce the granules, which were then allowed to dry at 25 °C for 24 h. The same method was used to produce pellets without the fungicide, that is without CHX. The pellets were then cut in approximately equal sizes and stored in a freezer inside plastic pots until they were required (adapted methodology) [4].

#### 2.1.2. Observation Box

The *Atta sexdens rubropilosa* Forel colonies were raised in the laboratory and used when they reached an average volume of 100 cm^3^ (volume of the ants, brood and fungus garden). After its growth, the garden fungus, as well as workers, brood and queen were transferred to glass boxes with a volume of 144 cm^3^, which was closed with a clear glass cover, to allow observation of behaviors carried out in the colony. The glass box was connected to two more chambers, one for foraging of the supplied plants and another for garbage deposition. The connection was made by transparent hoses (inner diameter: 12.7 mm) and wall thickness: 2.0 mm, length: 5 cm) that allowed ants to move between chambers.

#### 2.1.3. Experiment 1

We carried out eight hours of observations in each of the four colonies in order to record the behaviors performed in the preparation and incorporation of the citrus pulp pellets into the fungus garden. The colonies received CHX-pellets during 7 days followed by CHX-free pellets for the next 7 days, 14 days of observation in total. We performed two hours of observation per day until reaching the eight hours of each treatment. The observations were made at a time interval of ten minutes each, and the behaviors related to the citrus pulp pellets’ processing, mutual cleaning and self-cleaning were counted. The observation system was a scanning type with free observation, without stereoscopic finalization, in which a group of individuals is scanned quickly and the behavior of each one is recorded at regular intervals. The behaviors performed by the workers of *Atta sexdens rubropilosa* during the processing and incorporation of the citrus pulp pellets in the fungus garden were classified, and afterwards, the frequencies of the occurrence of these acts and their percentages were calculated.

#### 2.1.4. Experiment 2

We compared the two experimental groups, each with four colonies, in order to study the CHX effect on the colonies: (a) four colonies received CHX pellets during 14 days; and (b) four colonies receive pellets without CHX during 14 days. The variables studied were: the pellets’ transportation time to the fungus garden, according to the methodology referred to in Experiment 1, along with the eight hours of observation; pellets to the waste chamber, assessed by the amount of pellets returned 24 h after their supply; waste production, measured by the dry weight of the material deposited in the waste chamber 24 h after the pellet supply; and workers’ mortality, measured by the number of ants deposited in the waste chamber 24 h after the pellet supply.

#### 2.1.5. Statistical Analysis

Behavior frequency data were analyzed in two ways: (a) comparing behaviors in the first seven days (CHX pellets) to the seven subsequent days (without CHX) through a G test; and (b) comparing all behavioral acts in the 14-day observation through the Kruskal-Wallis test (non-parametric ANOVA, given the non-normal distribution of data) and by Student-Newman-Keuls post-test (5% significance).

The variables carrying orange pellets, pellet removal to the waste chamber, waste production and worker mortality were compared between both the colonies that received CHX pellets and those pellets without CHX using the Student’s *t*-test (5% significance). These same variables were compared in the initial seven days (CHX pellets) with a subsequent seven days (without CHX) through Student’s *t*-test (5% significance).

## 3. Results

### 3.1. Experiment 1

**Figure 1:**
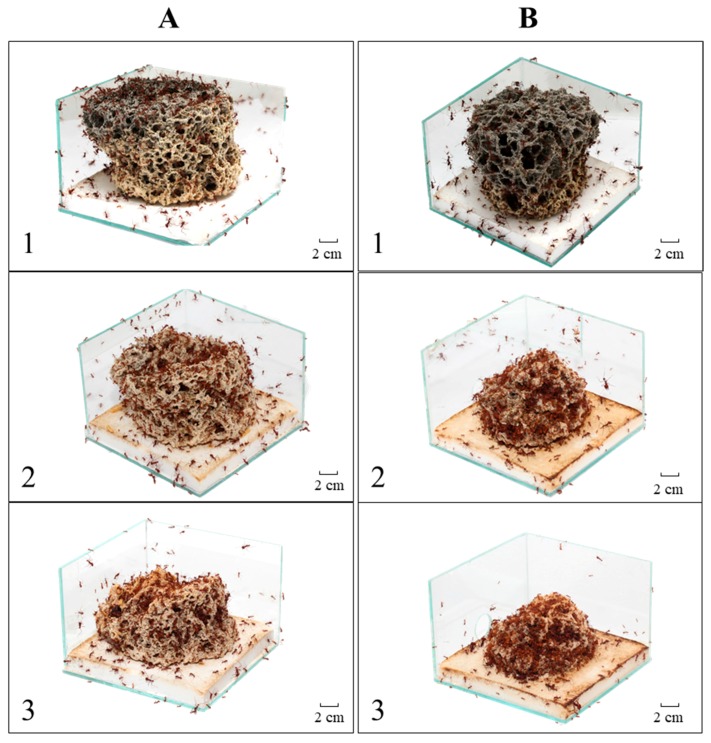
Fungus garden during Experiment 1. (**A**) Colony A; and (**B**) Colony B. Photo 1: healthy fungus; Photo 2: seventh day of pellets with cycloheximide (CHX); and 3: 14th day of the experiment.

As expected, we found differences in the fungus structure (Figure 1) and in the behavioral frequencies when we compared the initial seven days (CHX pellets) to the subsequent seven days (without CHX). The behaviors showed a normal frequency in the first contact with the fungicide; there were no sudden changes in behavior in relation to the other days. The following differences were observed: depositing orange pellets on the fungus garden surface (G test, G = 515.23, df = 6, *p* < 0.0001), pellet licking (G test, G = 452.40, df = 6, *p* < 0.0001), keeping pellets in the fungus garden (G test, G = 428.09, df = 6, *p* < 0.0001), chopping pellets (G test, G = 980.47, df = 6, *p* < 0.0001), chewing fragments after chopping them (G test, G = 792.42, df = 6, *p* < 0.0001), incorporating the fragments into the fungus garden (G test, G = 416.56, df = 6, *p* < 0.0001), licking the surface of newly-incorporated fragments (G test, G = 2606.5, df = 6, *p* < 0.0001), deposition of hyphae tufts on the incorporated fragments (G test, G = 215.10, df = 6, *p* < 0.0001), cleaning between ants (G test, G = 1574.08, df = 6, *p* < 0.0001) and transporting fragments to the waste chamber (G test, G = 147.85, df = 6, *p* < 0.0001) (Table 1). We also detected significant differences when we compared the behavioral frequencies between each other (Kruskal–Wallis test, H = 87.67, df = 9, *p* < 0.001).

### 3.2. Experiment 2

**Figure 2:**
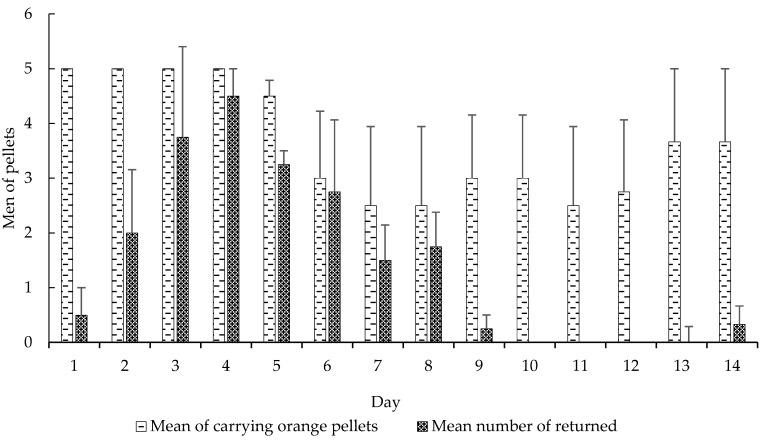
Mean (±SE) of carrying orange pellets regarding the mean number of returned pellets. Days using CHX pellets and days using pellets without CHX. Experiment 1.

**Figure 3:**
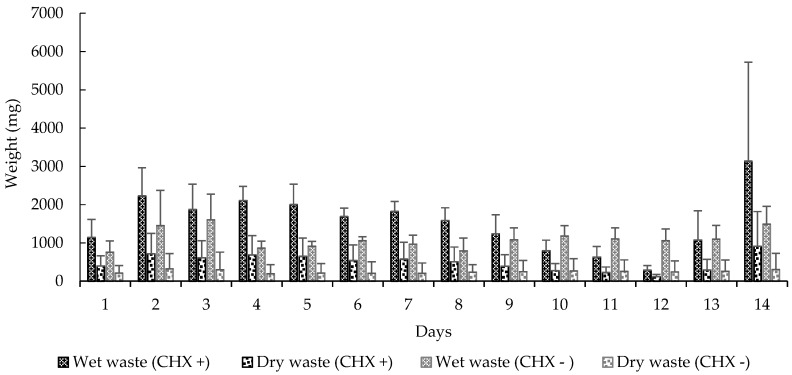
Mean (±SE) weight of waste deposited at the waste chamber by the workers. CHX +: With cycloheximide; CHX −: Without cycloheximide. Experiment 2.

**Figure 4:**
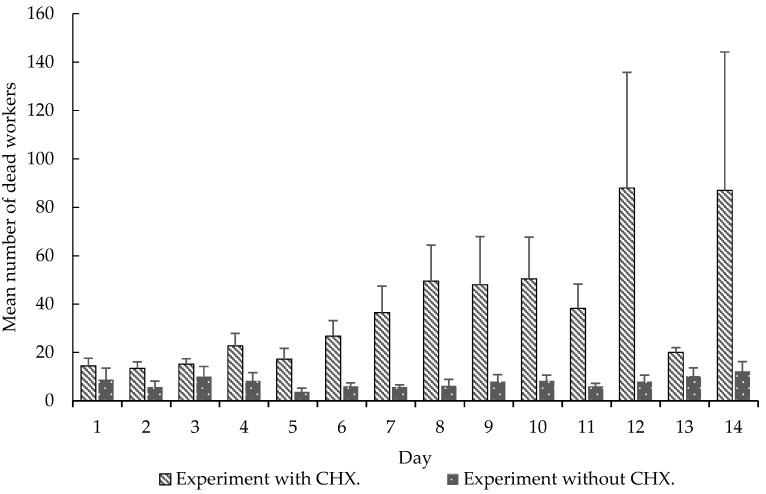
Mean (±SE) number of dead worker over 14 days. Experiment with CHX and experiment without CHX. Experiment 2.

We also observed significant differences in these variables: carrying orange pellets (*t*-test, *t* = −5.0898, df = 13, *p* < 0.0001), pellet removal to the waste chamber (*t*-test, *t* = 3.5664, df = 13, *p* < 0.0001) (Figure 2), amount of wet (*t*-test, *t* = 2.2051, df = 13, *p* < 0.05) and dry waste (*t*-test, *t* = 4.0943, df = 13, *p* < 0.001) (Figure 3) and, finally, the mortality of small (*t*-test, *t* = 5.0370, df = 13, *p* < 0.001) and mid-sized workers (*t*-test, *t* = 4.5115, df = 13, *p* < 0.001) in colonies that received CHX pellets compared to colonies that received pellets without CHX (Figure 4). We observed and analyzed the average time spent in carrying orange pellets with and without CHX, which showed an increase in the time spent in this transportation from the third day onward (Table 2).

When we compare these variables in the initial seven days (CHX pellets) to the seven subsequent days (pellets without CHX), we observed significant differences in the carrying of orange pellets (*t*-test, *t* = 3.7146, df = 6, *p* < 0.05), removal of pellets to the waste chamber (*t*-test, *t* = 3.9375, df = 6, *p* < 0.001), wet (*t*-test, *t* = 2.3567, df = 6, *p* < 0.0001) and dry waste production (*t*-test, *t* = 2.5205, df = 6, *p* < 0.05) and, finally, mortality of small (*t*-test, *t* = 3.8907, df = 6, *p* < 0.001) and mid-sized workers (*t*-test, *t* = 3.2122, df = 6, *p* < 0.05).

## 4. Discussion

Undoubtedly, CHX adversely affected the colonies of leaf-cutting ants (Figure 1), in particular the workers’ behavior during the transportation, preparation and incorporation of pellets into the fungus garden. Our transportation result corroborates that observed by Ridley et al., in which an initial transportation occurs with a subsequent rejection of the citrus pellets, although the author did not study the ants’ behavior during the symbiotic cultivation. These observations of the ants’ behavior during the pellet processing clearly demonstrate their decision to change their foraging behavior. This behavioral change has already been studied regarding the physical resistance of the foraging material as a post-selection criterion within the nest. However, post-selection was only a behavioral change [21,22,23], whereas in our study, we could attribute either a possible fungus-worker communication [5] or just a response to the CHX toxicity to the workers.

During the 14 days, the workers’ behavioral frequencies varied according to the presence of CHX in the pellets (Table 1). On the first day of delivery of the pellets, the behaviors showed a normal frequency, that is in the first contact with the fungicide, there were no sudden changes in behavior compared to other days. After seven days of supplying CHX pellets, there were changes in the behavioral frequencies, mainly an increase in the frequency of pellet licking, as well as cleaning among the ants, which was observed from the third day of the experiment. This increase in frequency, already observed in other studies [24,25], may suggest that there is an increase in pellet preparation before incorporation, since some leaf-cutting ants have antifungal and antibiotic properties in their saliva that can promote asepsis in pellets for their incorporation [26]. The same may be mentioned about the increase of the cleaning among the ants, with a possible increase in asepsis among the contaminated workers.

The fact that the ants do not detect this fungicide is analogous to the insecticides currently used in their control [27], that is there is a transport of the toxic baits with an active ingredient of delayed action, taking days for the colony contamination as a whole. In some cases, baits are returned to the exterior of the nest [28], similar to the pellet return rate observed in our results (Figure 2). However, certain plants may also contain deleterious substances to the fungus, promoting their non-transport or even rejection [29]. These substances are detected by the ants, probably through an allomone produced by the fungus, which acts as a negative reinforcement to them [4]. Once the colony has suffered such an effect, the plant is rejected for days, or even weeks [29]. Thus, not only materials with substances harmful to the fungus are rejected or discarded, but so are materials unsuitable for fungus garden cultivation [22,23]. Some authors suggest that learning occurs through the refusal by the workers to forage a palatable plant, even long-term memory [10], although the mechanism has not yet been elucidated [12]. Recently, it has been found that this long-term memory of avoidance is associated with a transient increase of the synaptic complex of the mushroom body in leaf-cutter ants [11]. In our study, it was possible to observe an increase in the pellets’ transport time to the fungus chamber after the third day (Figure 4). Possibly, this long-term memory was formed to avoid this material, after the workers detected the damage caused by the substrate incorporated into the colony. This may have led to an increase in the time taken to take the pellets into the fungus chamber (Table 2).

Although this long-term memory is used to explain the dynamics of foraging and plant selection by leaf-cutting ants [5, Ridley, et al. 4], used CHX as an agent that induces colonial collapse. The aforementioned authors exclude the possibility of CHX causing toxicity to workers, besides acting on the symbiontic fungus. CHX is toxic to many insects, such as flies, crickets, butterflies and beetles, among others, with diverse modes of action, such as long-term memory formation, toxicity of resistant genotypes, altered morphology, altered protein levels and neuronal destruction [13,14,15,16,17,18,19,20]. Our study showed a high mortality of the workers that received the CHX pellets (Figure 4), differing significantly from the control group without CHX.

## 5. Conclusions

We conclude that cycloheximide alters the behavioral frequencies of leaf-cutting ant workers when homogenized in pellets, with the occurrence of increased frequencies of licking the pellets and cleaning among the workers. In addition, there is a high mortality of the ants and the observation of damage in the fungus garden in the course of cycloheximide incorporation.

## Figures and Tables

**Table 1:** Mean frequency (±SE) of behavior acts of workers. Days using CHX pellets and days using pellets without CHX. 1: Depositing orange pellets on the fungus garden surface; 2: pellet licking; 3: keeping pellets in the fungus garden; 4: pellet chopping; 5: chewing the fragments after chopping; 6: incorporated fragments into the fungus garden; 7: licking the newly-incorporated fragments; 8: depositing hyphae tufts on the incorporated fragments; 9: ants’ cleaning; and 10: transporting fragments to the waste chamber. Experiment 1.

Mean of Behaviors														
Behaviors	1 Day	2 Day	3 Day	4 Day	5 Day	6 Day	7 Day	8 Day	9 Day	10 Day	11 Day	12 Day	13 Day	14 Day
**1**	2.5 ± 1.0	34.8 ± 16.6	83 ± 31.6	51.3 ± 6.0	64.5 ± 28.8	29.3 ± 21.0	16.8 ± 15.1	42.8 ± 30.4	40 ± 31.1	56 ± 43.7	11.8 ± 6.8	6.0 ± 3.5	5.7 ± 2.5	5.7 ± 1.8
**2**	268.75 ± 29.0	389.3 ± 63.6	543.8 ± 112.3	601.3 ± 88.3	438.5 ± 60.6	314.8 ± 136.3	163.8 ± 95.2	262.5 ± 154.6	463 ± 216.5	397.8 ± 168.8	303.8 ± 101.0	290.8 ± 67.4	308 ± 72.6	258.3 ± 101.2
**3**	13.75 ± 2.3	7 ± 3.5	4.8 ± 1.7	4.5 ± 2.6	1.3 ± 0.9	0.0	0.0	0.0	0.3 ± 0.3	0.3 ± 0.3	0.3 ± 0.3	0.8 ± 0.5	1.3 ± 0.8	0.7 ± 0.6
**4**	39.75 ± 7.0	38 ± 26.0	18 ± 14.3	7.5 ± 4.5	0.3 ± 0.3	0.5 ± 0.5	0.3 ± 0.3	1.3 ± 1.3	6.3 ± 3.7	28.5 ± 17.0	48.5 ± 28.1	32.8 ± 19.6	74.7 ± 35.6	67.7 ± 29.3
**5**	30 ± 3.7	27.3 ± 16.0	16.8 ± 12.8	10.5 ± 6.3	0.0	0.0	1.0 ± 1.1	0.8 ± 0.8	5 ± 3.0	34.3 ± 22.0	43 ± 25.5	36.3 ± 0.5	57.7 ± 25.0	73.3 ± 31.8
**6**	47 ± 12.8	5 ± 3.0	11.8 ± 9.6	4.8 ± 3.3	0.0	0.0	0.5 ± 0.5	0.3 ± 0.3	0.3 ± 0.3	7.3 ± 5.4	7 ± 4.4	9.3 ± 6.6	16.7 ± 7.2	12.3 ± 5.3
**7**	266.25 ± 65.4	36.3 ± 28.3	21.8 ± 12.6	10.5 ± 6.1	0.0	0.0	1.5 ± 1.5	0.5 ± 0.5	0.0	3.3 ± 2.1	100.3 ± 60.2	83 ± 4.2	136 ± 62.0	111.7 ± 54.5
**8**	11.25 ± 2.9	3.8 ± 2.4	4 ± 2.6	2.3 ± 1.3	0.0	0.0	0.0	0.0	0.0	0.0	3.0 ± 1.8	4.8 ± 2.8	11.3 ± 5.8	8.7 ± 3.9
**9**	226.75 ± 76.6	245 ± 75.8	287.3 ± 82.8	377 ± 109.4	473.3 ± 135.6	517.3 ± 201.4	590.3 ± 162.9	593 ± 127.5	505 ± 169.6	609 ± 57.7	591.8 ± 84.9	545.3 ± 122.8	441 ± 53.9	526 ± 132.0
**10**	37.25 ± 6.7	42.3 ± 11.8	45.3 ± 11.9	60.3 ± 8.2	40.3 ± 5.2	71 ± 64.6	43.8 ± 2	38 ± 7.1	32.3 ± 6.1	16.8 ± 1.1	10.8 ± 3.8	7 ± 3.8	25.7 ±11.0	43 ± 13.0

**Table 2:** Mean (±SE) number of pellets carried and mean time spent carrying them during the experiment. With cycloheximide (CHX +); Without cycloheximide (CHX −). Experiment 2.

Day	Mean Number of Pellets		Mean Time (min) Spent	
	CHX +	CHX −	CHX +	CHX −
1	5.0 ± 0.0	5.0 ± 0.0	5.5 ± 0.5	5.0 ± 0.0
2	5.0 ± 0.0	5.0 ± 0.0	14.3 ± 8.6	5.0 ± 0.0
3	5.0 ± 0.0	5.0 ± 0.0	24.8 ± 12.2	5.0 ± 0.0
4	3.8 ± 1.3	5.0 ± 0.0	21.5 ± 8.5	5.0 ± 0.0
5	3.8 ± 0.9	5.0 ± 0.0	246.5 ± 134.8	5.0 ± 0.0
6	3.0 ± 1.2	5.0 ± 0.0	260.3 ± 127.3	5.0 ± 0.0
7	2.5 ± 1.4	5.0 ± 0.0	266.8 ± 123.2	5.0 ± 0.0
8	3.8 ± 1.3	5.0 ± 0.0	138.5 ± 114.9	5.0 ± 0.0
9	3.0 ± 1.2	5.0 ± 0.0	252 ± 131.6	5.0 ± 0.0
10	2.5 ± 1.4	5.0 ± 0.0	124 ± 118.7	5.0 ± 0.0
11	2.5 ± 1.4	5.0 ± 0.0	125.8 ± 118.1	5.0 ± 0.0
12	2.8 ± 1.3	5.0 ± 0.0	125.8 ± 118.1	5.0 ± 0.0
13	3.7 ± 1.3	5.0 ± 0.0	165 ± 157.5	5.0 ± 0.0
14	3.7 ± 1.3	5.0 ± 0.0	165 ± 157.5	5.0 ± 0.0
